# Management of desmoid tumors associated with familial adenomatous polyposis: a three-decade experience of a tertiary center in Brazil

**DOI:** 10.1590/0102-67202025000033e1902

**Published:** 2025-10-31

**Authors:** Amanda Pereira LIMA, Raquel Franco LEAL, Michel Gardere CAMARGO, Carlos Augusto Real MARTINEZ, João José FAGUNDES, Claudio Saddy Rodrigues COY, Maria de Lourdes Setsuko AYRIZONO

**Affiliations:** 1 Universidade Estadual de Campinas, Faculty of Medical Sciences, Department of Surgery, Colorectal Unit – Campinas (SP), Brazil.

**Keywords:** Desmoid Tumors, Adenomatous Polyposis Coli, Gardner Syndrome, Colorectal Neoplasms, Tumores Desmóides, Polipose Adenomatosa do Colo, Síndrome de Gardner, Neoplasias Colorretais

## Abstract

**Background:**

Aggressive fibromatosis, also known as desmoid tumor (DT), is a locally aggressive myofibroblastic neoplasm originating from deep soft tissues, characterized by an infiltrative growth pattern with a tendency for local recurrence. DTs account for 0.03% of all neoplasms, and cases associated with familial adenomatous polyposis (FAP) account for 5–15% of DTs.

**Aims:**

The aim of this study was to report the prevalence of DTs in patients operated on for FAP, describe the epidemiological profile, and evaluate the risk factors for tumor development, treatments performed, associated complications, and follow-up.

**Methods:**

This retrospective study assessed the medical records of patients with FAP who underwent surgery between 1990 and 2021 and developed DTs during follow-up.

**Results:**

In the study period, 147 patients with FAP were operated on; of these, 97 underwent total proctocolectomy with ileal-pouch anal anastomosis, 33 underwent total colectomy with ileorectal anastomosis (IRA), 14 underwent total proctocolectomy with terminal ileostomy, and three underwent total colectomy with partial proctectomy and low IRA using an ileal-pouch. A total of 26 patients (17.7%) developed DT; most were female (61.5%), were White (73.1%), and had a family history (84.6%). The most frequent complications were intestinal and ureteral obstructions. Long-term follow-up showed that six patients were free of disease, 14 were stable and undergoing drug therapy, four died due to complications of the disease, and two were lost to follow-up.

**Conclusions:**

The prevalence of DT tumor was relatively high and more commonly observed in patients with a family history of the tumor. The disease presented high rates of morbidity and mortality.

## ARTICLE HIGHLIGHTS

Desmoid tumor (DT) is an aggressive myofibroblastic neoplasm characterized by an infiltrative growth pattern with a tendency for local recurrence without metastatic potential.

The clinical symptoms of DTs depend on their location, size, number of lesions, and growth pattern. DTs can be classified into intra-abdominal, abdominal wall, and extra-abdominal.

FAP cases associated with DTs account for 5–15% of this type of tumor. They often have a more aggressive course and may present as more extensive, multifocal tumors, occurring up to 10 years earlier than sporadic ones.

The treatment of DTs is multimodal and depends on several factors, such as tumor size and location, symptoms, and lesion growth pattern. They include surgery, active monitoring, radiotherapy, chemotherapy, hormone therapy, nonsteroidal anti-inflammatory drugs, and ablation techniques.

## Summary

**CENTRAL MESSAGE:**

Aggressive fibromatosis, also known as desmoid tumor (DT), is a locally aggressive myofibroblastic neoplasm originating from deep soft tissues, characterized by an infiltrative growth pattern with a tendency for local recurrence without metastatic potential. DTs account for 0.03% of all neoplasms and <3% of soft tissue tumors in the general population. The incidence of DTs in European countries is 2–5 cases per one million inhabitants, with a mean age at diagnosis of 35 years. Cases associated with familial adenomatous polyposis (FAP) syndrome account for 5–15% of DTs.

**PERSPECTIVES:**

In this study, the prevalence of desmoid tumors (DTs) in patients operated on for familial adenomatous polyposis (FAP) was relatively high (17.7%) and comparable with that reported in the literature. DTs were more commonly observed in patients with a family history of the condition. Surgical treatment was the most prevalent, and morbidity and mortality rates were high. However, many questions remain regarding the optimal management of DTs. Although rare, the disease is characterized by a low prevalence but a high rate of complications, and currently, there are no standardized treatment protocols. Further studies are needed to obtain a better follow-up of these patients to enable increased survival through earlier diagnosis, more effective treatment strategies, and, where possible, prevention.

## VISUAL ABSTRACT

**Figure:**
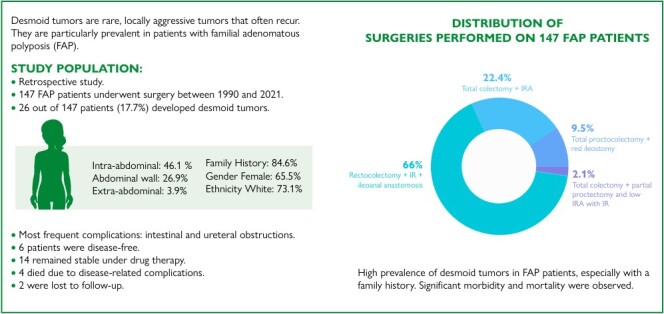

## RESUMO

**Racional:**

A fibromatose agressiva, também conhecida como tumor desmoide (TD), é uma neoplasia miofibroblástica localmente agressiva originária de tecidos moles profundos, caracterizada por um padrão de crescimento infiltrativo com tendência à recorrência local. Representam 0,03% das neoplasias, e os casos associados à polipose adenomatosa familiar (PAF) representam 5–15% dos TD.

**Objetivos:**

Relatar a prevalência de TD em pacientes operados por PAF, descrever o perfil epidemiológico e avaliar os fatores de risco para o desenvolvimento do tumor, tratamentos realizados, complicações associadas e acompanhamento.

**Métodos:**

Este estudo retrospectivo avaliou os prontuários médicos de pacientes com PAF que foram submetidos à cirurgia entre 1990 e 2021 e desenvolveram TD durante o acompanhamento.

**Resultados:**

No período do estudo, 147 pacientes com PAF foram operados: 97 foram submetidos à retocolectomia total e anastomose ileanal com reservatório ileal; 33, colectomias totais com anastomose ileorretal; 14, proctocolectomias totais com ileostomia terminal; e três, colectomia total com proctectomia parcial e anastomose ileorretal baixa com reservatório ileal. Vinte e seis pacientes (17,7%) desenvolveram TD; a maioria era do sexo feminino (61,5%), branca (73,1%) e tinha histórico familiar de TD (84,6%). As complicações mais frequentes foram obstruções intestinais e ureterais. O acompanhamento em longo prazo mostrou que seis pacientes estavam livres da doença, 14 estavam estáveis em tratamento medicamentoso, quatro morreram devido a complicações da doença e dois perderam o acompanhamento.

**Conclusões:**

A prevalência de TD foi relativamente alta e mais comumente observada em pacientes com histórico familiar do tumor. A doença apresentou altas taxas de morbidade e mortalidade.

## INTRODUCTION

 Aggressive fibromatosis (AF), also known as desmoid tumor (DT), is a locally aggressive myofibroblastic neoplasm originating from deep soft tissues, characterized by an infiltrative growth pattern with a tendency for local recurrence without metastatic potential^14^. The term "desmoid" was first used by anatomist and physiologist Johannes Muller in 1832 and is derived from the Greek word *desmos*, reflecting its tendon-like appearance upon histological evaluation^8,15^. 

 DTs account for 0.03% of all neoplasms and <3% of soft tissue tumors in the general population. The incidence of DTs in European countries is 2–5 cases per one million inhabitants, with a mean age at diagnosis of 35 years^15^. Cases associated with familial adenomatous polyposis (FAP) syndrome^6,25^ account for 5–15% of DTs. The first case of DTs occurring in association with FAP was reported in 1923 by Ralph W. Nichols^24^. 

 Somatic and germline inactivation of the *adenomatous polyposis coli* (APC) gene is a fundamental step in the molecular pathways involving DT formation. However, other environmental factors are involved, such as family history, previous abdominal surgery, female sex, pregnancy, and estrogen therapy^41^. Patients with FAP presenting with an APC 3 of codon 1444 mutation have a 12-fold higher risk of developing DTs^8,32^. 

 Radiological studies are essential for diagnosis, treatment, and follow-up. Ultrasonography (US), computed tomography (CT), and nuclear magnetic resonance (NMR) imaging are the main methods used. The treatment of AF is multimodal and depends on several factors, such as tumor size and location, symptoms, and lesion growth pattern^15^. Therapeutic options include surgery, active monitoring (watch and wait), radiotherapy (RT), chemotherapy (CTX), hormone therapy, and nonsteroidal anti-inflammatory drugs (NSAIDs), as well as ablation techniques (radiofrequency and cryoablation)^13,22 ,36^. 

 The objective of this study was to report the prevalence of DTs in patients operated on for FAP, describe the epidemiological profile, and evaluate the risk factors for the occurrence of DTs, treatments performed, associated complications, and long-term follow-up. 

## METHODS

 This retrospective study included patients operated on for FAP in the period from January 1990 to October 2021 at the Colorectal Surgery Unit of the Department of Surgery at the University of Campinas (Unicamp) and who subsequently developed DTs during follow-up. 

 The diagnosis of FAP was based on endoscopic and anatomopathological examination and family history of the disease; research on the APC gene mutation was not available in our unit. The surgical procedures performed for FAP were total colectomy with ileorectal anastomosis (IRA); total colectomy with partial proctectomy and low IRA with an ileal-pouch (IP); total proctocolectomy with ileal-pouch anal anastomosis (IPAA); and total proctocolectomy with end ileostomy. 

 A combination of clinical and radiological examinations was used to diagnose DTs, and the characteristics of the lesions were based on data from imaging methods, surgical descriptions, and anatomopathological studies of surgical specimens. The variables analyzed were age, sex, race, family history of DT, tumor location and size, number of lesions per patient, treatment modalities, complications, follow-up duration, type of colorectal surgery performed, and time between surgery and the appearance of DT. 

 The DT location was classified as intra-abdominal, extraabdominal, and abdominal wall. The measurement of DT size was based on pathological data and, when not operated on, reports of imaging tests. Data were entered into a Microsoft Excel spreadsheet, and all variables were analyzed. The association between the type of surgery and the occurrence of DTs was assessed using the chi-squared test; the test of proportions was used for comparison between the proportions of sex, race, and family history of DT. The mean age between sexes was compared using the Mann-Whitney test, and the significance level adopted was 5%. This study was approved by the Research Ethics Committee of the institution (CAAE no. 13411419.7.0000.5404). 

## RESULTS

 During the period studied, 147 patients underwent surgery for FAP, of which 97 underwent proctocolectomy with IPAA (66.0%), 33 had total colectomy with IRA (22.4%), 14 underwent total proctocolectomy with end ileostomy (9.5%), and three underwent total colectomy with partial proctectomy and low IRA anastomosis with an IP (2.1%) (Table 1).

**Table 1:** Surgeries performed for familial adenomatous polyposis (1990–2021).

Type of surgery	Number of surgeries (%)
Proctocolectomy and IPAA	97 (66.0)
Colectomy + IRA	33 (22.4)
Proctocolectomy + end ileostomy	14 (9.5)
Colectomy + partial proctectomy and low IRA with IP	3 (2.1)
Total	147 (100)

IPAA: ileal-pouch anal anastomosis; IRA: ileorectal anastomosis; IP: ileal-pouch.

 All surgeries were performed with laparotomic access. During follow-up, 26 patients developed DTs (17.7%). The prevalence of DTs in the proctocolectomy and IPAA group was 17.7%, in the total colectomy with IRA group was 21.2%, and in the total proctocolectomy group was 3.3%. Patients with an IP (total proctocolectomy with IPAA and total colectomy+partial proctectomy with an IP) showed a DT prevalence of 17.0%, and the group without IP (total colectomy+IRA and total proctocolectomy+terminal ileostomy) presented with a prevalence of 19.1%. There was no significant difference regarding the occurrence of DTs (Table 2). 

**Table 2:** Association between surgery with and without an ileal-pouch and the occurrence of desmoid tumor.

Surgery performed	Total number of patients	DT		p-value
		No (%)	Yes (%)	
Surgery with IP	100	83 (83.0)	17 (17.0)	0.931
Surgery without IP	47	38 (80.9)	9 (19.1)	
Total	147	121	26	

DT: desmoid tumor; IP: ileal-pouch.

 No significant association was observed between sex and DT or race and DT. However, the presence of a family history of the tumor was a substantial factor in the development of DTs (p<0.001) (Table 3). The mean age at DT diagnosis was 29.8 years: 27.1 years in women and 34.3 years in men, with no significant difference (p=0.057, p>0.05) (Table 3). 

**Table 3:** Characteristics of patients with desmoid tumor.

Characteristics		DT/total (%)	p-value
Sex			
	Female	16/86 (18.6)	0.899
	Male	10/61 (16.4)	
Race			
	White	19/117 (16.2)	0.522
	Non-white	07/30 (23.3)	
Family history of DT			
	Yes	22 (84.6)	<0.001
	No	04 (15.4)	
Mean age			
	Women	27.1 (16–39)	0.057
	Men	34.3 (20–57)	

DT: desmoid tumor

 The mean time between FAP surgery and DT diagnosis was 60.6 months, ranging from 8.9 to 238.4 months. Regarding tumor location, 12 patients (46.1%) had an intra-abdominal DT; seven (26.9%) had a DT in the abdominal wall; and one (3.9%) had an extra-abdominal tumor. Six patients (23.1%) simultaneously presented with multiple locations (Table 4). Among the rarest tumor sites, we found one in the pancreatic body, breast, and paravertebral region. The tumor size ranged from 1.5 to 20 cm. Regarding the number of lesions, patients had one to five tumors. 

**Table 4:** Desmoid tumor location in patients and surgical treatment.

Location	Total number (%)	Surgical treatment (%)	
		Yes	No
Intra-abdominal	12 (46.1)	6	6
Abdominal wall	7 (26.9)	7	0
Extra-abdominal	1 (3.9)	1	0
Intra-abdominal + abdominal wall	3 (11.5)	2	1
Intra-abdominal + extra-abdominal	2 (7.7)	2	0
Abdominal wall + extra-abdominal	1 (3.9)	1	0
Total	26 (100)	19 (73.1)	7 (26.9)

**Figure 1:**
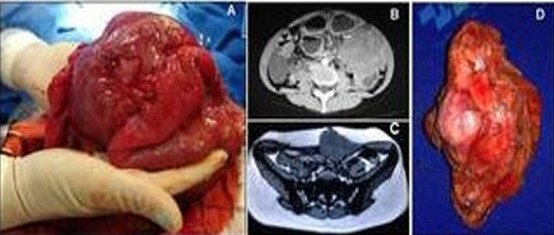
Aspects of desmoid tumor. **(A)** Intraoperative; **(B)** Computed tomography imaging; **(C)** Nuclear magnetic resonance imaging; **(D)** Surgical specimen.

 Most patients underwent surgical treatment (Figure 1); however, six patients with an intra-abdominal DT and one patient with both intra-abdominal and abdominal wall lesions could not undergo surgical resection of the tumor. 

 Several patients had intestinal subocclusion, and four were submitted for laparotomy due to obstruction: three due to tumor growth and one due to adhesions. Moreover, one patient was presented with perforation of the ileum due to the use of NSAIDs. Ureteral obstruction was observed in four patients, which required unblocking by passing a double-J stent. One patient presented with superior vena cava syndrome, and another presented with spinal cord compression due to tumor growth. 

 During follow-up, six patients who underwent surgical treatment were free of disease, 14 were stable and undergoing drug therapy, and two were lost to follow-up. Four patients died because of disease progression: three due to sepsis with an abdominal focus and one due to a urinary focus (Table 5). Most patients with unresectable tumors used NSAIDs combined with tamoxifen; five underwent CTX, and one patient required a combination of CTX and RT due to tumor recurrence in the chest wall. 

**Table 5:** Follow-up of patients with desmoid tumor.

Follow-up	n (%)
Free of disease	6 (23.1)
Stable with medical treatments	14 (53.8)
Died	4 (7.7)
Lost to follow-up	2 (15.4)
Total	26 (100)

## DISCUSSION

 The clinical symptoms of AF depend on its location, size, number of lesions, and growth pattern. Based on location, it can be classified as intra-abdominal (mesenteric or retroperitoneal tissues), abdominal wall, and extra-abdominal (muscles of the trunk, neck, or extremities)^15 ,21^. 

 The most common presentation of AF is a single, painless, round or oval-shaped tumor that is hard on palpation. In the case of parietal infiltration, it can lead to deformities; local invasion can cause pain, asthenia, paresthesia, and neuropathy^15^. These neoplasms have a slow, progressive growth and an excellent capacity for local invasion, and are non-metastatic. Furthermore, their symptoms are often attributed to compression of organs of the gastrointestinal tract, urinary, nervous, or vascular systems, leading to complications such as intestinal obstruction/perforation, fistula, and digestive hemorrhage, with a risk of death^5 ,8,33^. Sudden DT growth is usually attributed to liquefaction necrosis or abscess formation^8^. 

 FAP-associated DTs often have a more aggressive course and may present as more extensive, multifocal tumors, occurring up to 10 years earlier than sporadic ones^13^. Since prophylactic surgery has reduced the rate of mortality from colorectal cancer, intra-abdominal DTs have been the most common cause of death in these patients^4,8^. The mean time between surgery and DT diagnosis is usually 2–3 years; whether the age at the surgical procedure and the type of surgery performed influence the development of DTs remains unknown^1,33^. 

 Surgical trauma is a risk factor for the development of DTs, probably because the proliferative phase of healing leads to the formation of a pathogenic variant of the APC gene in the mesenteric fibro-adipose tissue, causing complete loss of this tissue and the formation of a tumor^35^. Since surgical history is the leading risk factor for DTs, prophylactic colectomy should be postponed, if possible, especially in young patients with a family history of DTs of >50% in the limbs, with a germline mutation at codon 1444, or with mutations in the 3’ region of APC^8^. 

 Although the literature states that abdominal surgery incites DT formation, there is no consensus on the real impact of factors associated with the surgical procedure, such as time, extent of resection, and surgical technique used^35^. In a meta-analysis involving eight studies and 1,072 patients with FAP (491 undergoing proctocolectomy with IPAA and 581 undergoing total colectomy with IRA), Xie et al.^40^ found a DT occurrence rate of 11.8% following IPAA and 9.5% fol lowing IRA, with no significant difference between the two groups (odds ratio [OR]: 0.95, 95% confidence interval [CI]: 0.55–1.63, p=0.85, p>0.05). Similarly, a study of 256 patients by Konishi et al.^18^ and another European multicenter study of 852 patients with DTs also did not identify an association between DTs and the type of FAP surgery. 

 On the other hand, a more recent retrospective cohort investigated whether IPAA surgery is more "desmoidogenic" compared to IRA, given that there is more significant mesenteric manipulation in the former compared to the latter. Sommovilla et al.^35^ observed a 3-fold higher occurrence of DTs in the group undergoing IPAA (43.6 and 14.5%, respectively; OR 6.5; 95%CI 3.2–13.1; p<0.001 and p<0.05). However, in a systematic review and meta-analysis including 20 studies and 6,452 FAP patients, Aelvoet et al.^2^ did not find any significant difference in DT incidence after IRA versus IPAA (OR 0.99, 95%CI 0.69–1.42) and after open versus laparoscopic surgery (OR 0.88; 95%CI 0.42–1.86). 

 Laparoscopic surgery appears to have a protective effect on the development of DTs. An observational study by Sinha et al.^33^, which included 112 patients undergoing total colectomy with IRA via either a laparoscopic (61.6%) or laparotomic route, verified that the risk of DT formation was 4% and 16%, respectively (p=0.043, p<0.05). Campos et al.^7^ observed that the risk of developing DTs was associated with female sex, the surgical timing of surgery, and previous reoperations. The type of surgery and surgical approach (open versus laparoscopic) did not affect the occurrence of DTs. 

 Data from the literature show that 5–16% of patients with FAP will develop DT, with the majority arising within 5 years after surgery for polyposis^8,13^. In the present study, the prevalence of DTs in patients operated for FAP was 17.9%, which is similar to that reported in the studies by Inoue et al.^16^ (15.8%) and Campos et al.^6^ (14.3%). 

 A meta-analysis conducted by Sinha et al.^33^ demonstrated that patients with FAP submitted to abdominal surgery are 3-fold more likely to develop DTs. However, DTs can also occur in the absence of previous abdominal surgery, with a prevalence of 4% at the time of FAP surgery^33^. Durno et al.^11^ conducted a study involving 887 FAP patients and 121 DTs to assess the association between age at surgery and the development of DTs, concluding that women had a 2.5-fold increased risk of developing the tumor when they had been through early surgery (at the age of 18 or younger) compared to women who had surgery in adulthood. In addition, in a retrospective analysis of patients diagnosed with DTs at the Mayo Clinic between 1976 and 1999, Fallen et al.^12^ identified 447 cases of the disease, of which 15.7% were associated with FAP. There was a female predominance in both FAP and sporadic DT cases. 

 The mean age at DT diagnosis in our study was 29.8 years (±8.8) years, and the development of DTs was earlier in women, with a mean age of 27.1 years, while in men, it was 34.3 years. In a meta-analysis, Slowik et al.^34^ reviewed 222 cases of DTs associated with FAP. They identified a mean age of 30.3 years, similar to that found in the present study and that by Quintini et al.^28^, who observed a mean age of 29.6 years in patients with intra-abdominal DT. 

 In our study, 69.2% of the patients had a positive family history of DTs. This is similar to the meta-analysis performed by Sinha et al.^33^, which identified a family history of DT as the leading risk factor for the development of these tumors. In particular, people with APC 3’ codon 1444 mutation are 12-fold more likely to develop DTs. Some studies have reported that mutations in the APC gene are associated with a 65% risk of developing DTs in the mesentery^8^. Since we do not have genetic testing, as in many services in this country, a family history of DTs is the main factor observed. These patients should have a more rigorous follow-up with physical examination and imaging methods, such as NMR and CT. 

 Intra-abdominal DTs can lead to severe complications with the potential risk of death, including enterocutaneous fistula, ureteral obstruction, intestinal ischemia, intestinal obstruction, and bowel perforation^30^. In the present study, intraabdominal DTs were the most frequent (46.1%), comparable to other studies^26,30^, followed by abdominal wall DTs (26.9%). In a retrospective study analyzing 303 patients with FAP from the Japanese Society for Colon and Rectal Cancer database, Inoue et al.^16^ found 41 cases of DTs, of which 61% were of intra-abdominal. 

 Small bowel obstruction is the most common complication of intra-abdominal DTs, occurring in up to 27–58% of patients. It may be caused by a direct effect of the mass due to tumor growth or by infiltration of the mesentery by tumor cells, which causes intestinal retraction and sclerosis of the mesenteric vessels, leading to ischemic stenosis^15^. In a retrospective study of 133 patients, Xhaja et al.^39^ found that 35% of patients with DTs presented with at least one episode of intestinal obstruction, with a predominance in women (64%), with an interval of 4.1 years between the initial surgery and the first sign of obstruction. 

 Despite its inability to generate distant metastases, the infiltrative behavior of DTs can also lead to ureteral obstruction. Walton et al.^38^ analyzed 158 patients with FAP and intraabdominal DTs and identified a 25% prevalence of ureteral complications. Women were predominantly younger than men, and 75% of patients required urological intervention, which included endoscopic unblocking (68%), nephrostomy (13%), and ureteral reimplantation (8%). 

 The present study identified a patient with a DT in the pancreatic body, a rare entity that CT or NMR can diagnose. Its radiological appearance can mimic a hypodense solid neoplasm such as pancreatic ductal adenocarcinoma or cystic pan creatic neoplasms^31^. Due to the unfavorable location, this patient did not undergo surgery and was treated with NSAIDs. As a result, the patient developed partial regression and stabilization of the lesion. 

 The occurrence of superior vena cava syndrome in a patient with a DT located in the chest wall was another rare event. This syndrome is due to obstruction of the superior vena cava, leading to decreased venous return of the head, neck, and extremities. This causes edema of the face, arms, and neck; dyspnea and cough; and engorgement of the cervical veins^20^. The patient underwent resection surgery; however, the tumor recurred, and he underwent RT and CTX. The lesion has remained stable to date. 

 In a retrospective study evaluating 99 patients with a histological diagnosis of abdominal wall and extra-abdominal DTs, Tsukamoto et al.^37^ found no significant difference in disease-free survival between local surgical treatment and a "watch-and-wait" approach, with the latter being more commonly used for larger tumors. In this study, we observed that local recurrence was not associated with compromised surgical margins. 

 Before the 2000s, the gold standard of DT treatment was surgery with microscopically negative margins, which is similar to the treatment of other soft tissue sarcomas. However, surgical resection can result in functional and esthetic impairment in patients with benign disease without preventing local recurrence^37^. Recurrence at 5 years can be as high as 25–60%, regardless of a positive resection margin^13^. 

 Surgery should not be indicated for large intra-abdominal DTs with extensive involvement of the small bowel, mesentery, and large vessels, due to the high risk of morbidity. Moreover, DTs, especially intra-abdominal DTs, tend to recur even after surgical resection, and a second procedure may be required in 75–85% of cases. In asymptomatic tumors of critical locations (such as the mesentery), a closely monitored follow-up can be adopted^17^. 

 When active treatment is required for DTs, surgery can be considered the first-line procedure, provided that the surgical morbidity is low, especially for tumors located in the abdominal wall. The objective is to achieve a wide resection with microscopically free margins (R0); however, a resection with microscopically compromised margins (R1) is accepted to avoid esthetic or functional impairment^9^. Patients with AF should be referred to centers with experience in treating this disease to prevent mutilating surgery whenever possible. It is strongly recommended that such procedures be performed by surgeons with substantial expertise. 

 Seven patients presented with unresectable disease at diagnosis: three are stable and undergoing drug therapy with NSAIDs and tamoxifen. A study conducted by Quast et al.^27^, evaluating 134 patients with DTs treated with sulindac and high doses of antiestrogens, showed regression or stabilization of the tumor in 85.1% of patients. 

 CTX should be considered for the treatment of aggressive DTs that do not respond to tamoxifen or NSAIDs^19^. In the present study, 60% of the patients submitted to CTX died, showing the aggressiveness of the DTs that require this kind of therapy. Weekly CTX regimens, including methotrexate and vinca alkaloids (vinblastine or vinorelbine), are preferred due to their low toxicity and clinical benefit in up to 80% of patients. In the case of symptomatic disease or anatomical sites requiring a rapid response, such as the head and neck, regimens with anthracyclines can be considered^15^. 

 Desurmont et al.^10^, in a study to assess the results of different treatment outcomes, analyzed 79 FAP patients with 149 DTs. Among these, 11 patients had only surgical treatment, 17 had only medical treatments, 31 had combined treatment, and 20 had no treatment with spontaneous DT regression or stabilization. They demonstrated that CTX was the most efficient treatment, with a response rate of 77%. The rate in other treatments was as follows: sulindac+tamoxifen – 50%, tamoxifen – 40%, imatinib – 36%, and sulindac – 28%. Among the 42 surgical procedures, an R0 resection was performed in 62%, with the absence of recurrence for 54% of patients. 

 Intra-abdominal DTs represent the leading cause of death in patients with FAP who undergo prophylactic colectomy/ proctocolectomy^19^. A Japanese retrospective series of 154 patients with FAP undergoing prophylactic surgery from 1981 to 2017 demonstrated a DT incidence of 13.9%^33^. Furthermore, there was a higher prevalence in patients operated on after the 2000s (p=0.028, p<0.05), in which patients underwent more diagnostic tests, including CT, which probably contributed to higher DT diagnosis^33^. 

 Regarding mortality, a multivariate analysis by Quintini et al.^28^ reported that a tumor size >10 cm (OR 1.44), severe pain/opioid dependence (OR 2.22), and need for parenteral nutrition (OR 3.29) were negative factors for survival. In the present analysis, tumor size also showed a correlation with mortality. 

 DTs in FAP patients generally require evaluation by a multidisciplinary team, with several different treatment options^3,29^. However, surgical resection remains acceptable in selective cases where negative margins can be obtained with low morbidity and mortality^23^. 

## CONCLUSIONS

 Although the present study is based on a retrospective analysis of a relatively low number of patients, the prevalence of DTs in patients operated on for FAP was relatively high (17.7%) and comparable with that reported in the literature. It was more commonly observed in patients with a family history of DT. Surgical treatment was the most prevalent, and morbidity and mortality rates were high. However, many questions remain regarding the optimal management of DTs. Although rare, the disease is characterized by a low prevalence but a high rate of complications, and currently, there are no standardized treatment protocols. Further studies are needed to obtain a better follow-up of these patients to enable increased survival through earlier diagnosis, more effective treatment strategies, and, if possible, prevention. 

## Data Availability

The information regarding the investigation, methodology, and data analysis of the article is archived under the responsibility of the authors.
